# Supplementary material for: Impact of diabetes self-management, diabetes management self-efficacy and diabetes knowledge on glycemic control in people with Type 2 Diabetes (T2D): A multi-center study in Thailand
Source: PLoS One. 2020 Dec 31;15(12):e0244692. doi: 10.1371/journal.pone.0244692 (PMC7774854; doi:10.1371/journal.pone.0244692)
Supplement: S1 File — (PDF) [file pone.0244692.s001.pdf]

ENGLISH VERSION OF THE QUESTIONAIRRE

ID Code: _ _ _ _

This questionnaire consists of two parts: (1) Personal information (Demographics); and (2) Questions that will allow us to rate your diabetes self-management, diabetes management self-efficacy and diabetes knowledge.

Please check (ADD TICK) any box, or where appropriate, write answers in the spaces provided. Please attempt to answer all question.

(PART 1: Demographics)

1. Gender: ☐ Male; ☐ Female

2. Marital status:

- ☐ Single,
- ☐ Married,
- ☐ Widow, Divorced or Separated

3. Age …. years

4. Education:

- ☐ Unschooled,
- ☐ Primary,
- ☐ High,
- ☐ Bachelor,
- ☐ Master,
- ☐ Higher than masters

5. Religon:

- ☐ Buddhist,
- ☐ Muslim,
- ☐ Christian,
- ☐ Other: please specify …..

6. Family income:

- ☐ <5000 THB
- ☐ 5,000-9,999THB
- ☐ 10,000-14999THB
- ☐ 15,000-19,999THB
- ☐ 20,000-24,999THB
- ☐ >=25000 THB

7. Weight ….. kg

8. Height ….. cm

9. Time since Type 2 Diabetes diagnosis …… years

10. Family history of Type 2 diabetese

- No,
- Yes

If yes, Relationship (e.g. Mother, Brother)

11. Do you have any other diseases? If yes, please indicate any that apply (i.e. can be more than 1).

- Dyslipidemia
- Hypertension
- Heart disease
- Kidney disease
- Other, please specify

12. Are you being treated for your diabetes. What treatment are you receiving?

- No medicine
- Oral medicine
- Insulin
- Both oral medicine and insulin

13. Smoking:

- Current,
- Previous,
- Never

14. Alcohol:

- Current,
- Previous,
- Never

DIABETES MANAGEMENT SELF-EFFICACY

Please circle the number that best represents your confidence. The numbers range from 1 = least confident to 5 = most confident.

I am confident that:

1. I am able to check my blood sugar if necessary.

   1 2 3 4 5

2. I am able to decrease my blood sugar when the sugar level is too high (e.g., eat different foods)

   1 2 3 4 5

3. I am able to increase my blood sugar when the sugar level is too low (e.g., eat different foods).

   1 2 3 4 5

4. I am able to choose the foods that are best for my health.

   1 2 3 4 5

5. I am able to choose different foods and maintain a healthy eating plan.

   1 2 3 4 5

6. I am able to control my body weight and maintain it within the ideal weight range.

   1 2 3 4 5

7. I am able to examine both of my feet (e.g., for cuts or blisters).

   1 2 3 4 5

8. I am able to do enough physical activity (e.g., walking, aerobic exercise, and stretching exercises).

   1 2 3 4 5

9. I am able to maintain my eating plan when I am ill.

   1 2 3 4 5

10. I am able to follow a healthy eating plan most of the time.

    1 2 3 4 5

11. I am able to do more physical activity if the doctor advises me to do so

    1 2 3 4 5

12. When doing more physical activity, I am able to adjust my eating plan.

    1 2 3 4 5

13. I am able to follow a healthy eating plan when I am away from home.

    1 2 3 4 5

14. I am able to choose different foods and maintain my eating plan when I am away from home.

1 2 3 4 5

15. I am able to follow a healthy eating plan during traditional ceremonies.

1 2 3 4 5

16. I am able to choose different foods and maintain a healthy eating plan when I am eating at a party

1 2 3 4 5

17. I am able to maintain my eating plan when I am feeling stressed or anxious.

1 2 3 4 5

18. I am able to visit my doctor four times a year to monitor my diabetes

1 2 3 4 5

19. I am able to take my medication as prescribed.

1 2 3 4 5

20. I am able to maintain my medication when I am ill.

1 2 3 4 5

DIABETES SELF-MANAGEMENT:

The questions below ask you about your diabetes self-care activities during the pas t7 days. If you were sick during the past 7days, please think back to the last 7 days that you were not sick.

Diet

How many of the last SEVEN DAYS have you followed a healthful eating plan?

0 1 2 3 4 5 6 7

On average, over the past month, how many DAYS PER WEEK have you followed your eating plan?

0 1 2 3 4 5 6 7

On how many of the last SEVEN DAYS did you eat five or more servings of fruits and vegetables?

0 1 2 3 4 5 6 7

On how many of the last SEVEN DAYS did you eat high fat foods such as red meat or full-fat dairy products?

0 1 2 3 4 5 6 7

Exercise

On how many of the last SEVEN DAYS did you participate in at least 30 minutes of physical activity? (Total minutes of continuous activity, including walking).

0 1 2 3 4 5 6 7

On how many of the last SEVEN DAYS did you participate in a specific exercise session (such as aerobic dance, jogging, walking, biking) other than what you do around the house or as part of your work?

0 1 2 3 4 5 6 7

Blood Sugar Testing

On how many of the last SEVEN DAYS did you test your blood sugar?

0 1 2 3 4 5 6 7

On how many of the last SEVEN DAYS did you test your blood sugar the number of times recommended by your health care provider?

0 1 2 3 4 5 6 7

Foot Care

On how many of the last SEVEN DAYS did you check your feet?

0 1 2 3 4 5 6 7

On how many of the last SEVEN DAYS did you inspect the inside of your shoes?

0 1 2 3 4 5 6 7

Smoking

Have you smoked a cigarette—even one puff—during the past SEVEN DAYS?

No

Yes

If yes, how many cigarettes did you smoke on an average day?

Number of cigarettes:_______

Which of the following medications for your diabetes has your doctor prescribed?

Please check all that apply.

□ a An insulin shot 1 or 2 times a day.

□ b An insulin shot 3 or more times a day.

□ c Diabetes pills to control my blood sugar level.

□ d Other (specify):

□ e I have not been prescribed either insulin or pills for my diabetes.

Diet

On how many of the last SEVEN DAYS did you space carbohydrates evenly through the day?

0 1 2 3 4 5 6 7

Medications

On how many of the last SEVEN DAYS, did you take your recommended diabetes medication?

0 1 2 3 4 5 6 7

On how many of the last SEVEN DAYS did you take your recommended insulin injections?

0 1 2 3 4 5 6 7

On how many of the last SEVEN DAYS did you take your recommended number of diabetes pills?

0 1 2 3 4 5 6 7

Foot Care

On how many of the last SEVEN DAYS did you wash your feet?

0 1 2 3 4 5 6 7

On how many of the last SEVEN DAYS did you soak your feet?

0 1 2 3 4 5 6 7

On how many of the last SEVEN DAYS did you dry between your toes after washing?

0 1 2 3 4 5 6 7

DIABETES KNOWLEDGE:

There is only one correct answer for each question. If you know the answer, circle the letter in front of it. If you don't know the answer, circle the letter in front of "I don't know".

1. The usual cause of type 2 diabetes is:

    a) Eating too much sugar and other sweet foods

    b) Lack effective insulin in the body

    c) Failure of the kidneys to control sugar in the urine

    d) I don't know

2. In untreated diabetes the blood sugar is usually:

    a) Normal

    b) Increased

    c) Decreased

    d) I don't know

3. The NORMAL range for blood glucose is

    a) 2.8 mmol/l

    b) 6.1 mmol/l

    c) 7.0 mmol/l

    d) I don't know

4. Which of the following health problems is usually NOT complication of diabetes

    a) Kidney disease

    b) Eye problems

    c) Lung problems

    d) All the above

    e) I don't know

5. Which of the following is true?

   a) It does not matter if my diabetes is not fully controlled, as long as I do not have a coma

   b) It is best to show some sugar in the urine in order to avoid hypoglycemia

   c) Poor control of diabetes could result in a greater chance of complications later

   d) I don’t know

6. The key to the control of diabetes is:

   a) The balance between regular amounts of insulin/tablets, food and exercise

   b) The maintenance of a low level of sugar in the urine in order to prevent hypoglycemia

   c) A high-protein, high fiber diet

   d) I don’t know

7. People with diabetes should:

   a) Have their food cooked separately from that of the family

   b) Eat the same foods as the same time each day

   c) Vary their diet by substituting different foods correctly from the diet exchange list

   d) I don’t know 8. In general, fit patients with diabetes should exercise for

8. In general, fit patients with diabetes should exercise for

   a) 1 hour once a week

   b) 20 to 30 minutes 3 to 5 times a week

   c) 1 hour every day

   d) I don’t know

9. The general effect of exercise is to:

a) Lower the blood sugar level

b) Raise the blood sugar level

c) Increase sugar in the urine

d) I don’t know

10. Rice is mainly:

a) Protein

b) Carbohydrate

c) Fat

d) I don’t know

11. You can eat as much as you like of which of the following foods:

a) Apples

b) Celery

c) Meat

d) I don’t know

12. Self-monitoring of blood glucose is:

a) The key to determining the right amount of medication

b) Important to see the effect of diabetes control such as diet and exercise

c) Both a and b

d) I don’t know

13. People with diabetes should take good care of their feet because:

a) After a long period of time, injecting insulin into the legs may cause swelling of the feet

b) Flat feet are commonly associated with diabetes

c) Older people with diabetes may have poor circulation of the blood in this area

d) I don’t know

14. The action of diabetes pills:

a) Lower blood sugar

b) Increase insulin secretion

c) Increase insulin sensitivity

d) All above

e) I don’t know

ENGLISH VERSION OF MEDICAL RECORD COMPONENT

ID Code □□□□

Result of laboratory and complications (Specific research).

1. HbA1C Result……………….mg/dl

   Date (DD/MM/YYYY)…./…./….

2. LDL –cholesterol Result……………….mg/dl

   Date (DD/MM/YYYY)…./…./….

3. HDL-cholesterol Result……………….mg/dl

   Date (DD/MM/YYYY)…./…./….

4. Triglyceride Result……………….mg/dl

   Date (DD/MM/YYYY)…./…./….

5. Blood pressure

   a. Systolic BP Result……………….mmHg

   b. Diastolic BP Result……………….mmHg

   Date (DD/MM/YYYY)…./…./….

6. Chronic complications

   □ No

   □ Yes

   If yes, please √in □ (More than one answer)

- ☐ Cerebrovascular Accident; CVA
- ☐ Cerebral Infraction
- ☐ Ischemic Stroke
- ☐ Stroke, Not specify
- ☐ Cerebral Hemorrhage
- ☐ Transient Ischemic Attack; TIA
- ☐ Angina pectoris
- ☐ Congestive Heart Failure; CHF
- ☐ Myocardial Infraction; MI included Ischemic Heart Disease; IHD
- ☐ Coronary Revascularization
- ☐ Peripheral Arterial Disease, PAD
- ☐ Neuropathy (Painful neuropathy, Autonomic neuropathy, neurogenic bladder)
- ☐ Renal Insufficiency (CRI, CRF, CKD, ESRD)
- ☐ Diabetes Nephropathy; DN
- ☐ Diabetes Retinopathy; DR
- ☐ Other complications, please specify……………………
