# Supplementary material for: Impact of diabetes self-management, diabetes management self-efficacy and diabetes knowledge on glycemic control in people with Type 2 Diabetes (T2D): A multi-center study in Thailand
Source: PLoS One. 2020 Dec 31;15(12):e0244692. doi: 10.1371/journal.pone.0244692 (PMC7774854; doi:10.1371/journal.pone.0244692)
Supplement: S2 File — (PDF) [file pone.0244692.s002.pdf]

แบบสอบถาม

ID Code □□□□

คำอธิบาย : แบบสอบถามนี้ประกอบด้วยสองส่วน คือ ส่วนที่ 1 ข้อมูลส่วนบุคคลของผู้ตอบแบบสอบถาม ส่วนที่ 2 เป็นเครื่องมือสำหรับการประเมินการรับรู้ความสามารถตนเอง การจัดการตนเอง และความรู้ของผู้ป่วยเบาหวานชนิดที่ 2

กรุณาทำเครื่องหมาย √ ลงในช่อง □ หรือเขียนคำตอบลงใน ....

ส่วนที่ 1 ข้อมูลส่วนบุคคล

1. เพศ □ ชาย □ หญิง

2. สถานภาพการสมรส □ โสด □ แต่งงาน □ หม้าย □ หย่า □ แยก

3. อายุ ปี ....

4. ระดับการศึกษา □ ไม่ได้เรียน □ ประถมศึกษา □ มัธยมศึกษา
□ ปริญญาตรี □ ปริญญาโท □ สูงกว่าปริญญาโท

5. ศาสนา □ พุทธ □ อิสลาม □ คริสต์ □ ศาสนาอื่น ๆ ระบุ............

6. รายได้รวมของครอบครัวต่อเดือน

□ ต่ำกว่า 4,999 บาท □ 5,000-9,999 บาท

□ 10,000-14,999 บาท □ 15,000-19,999 บาท

□ 20,000-24,999 บาท □ มากกว่า 25,000 บาท

7. น้ำหนัก ....กิโลกรัม

8. ส่วนสูง ....เซนติเมตร

9. ระยะเวลาที่ป่วยเป็นเบาหวาน.................. ปี

10. ประวัติการเจ็บป่วยด้วยโรคเบาหวานชนิดที่สองของสมาชิกในครอบครัว ☐มี ☐ไม่มี

ถ้ามีโปรดระบุความเกี่ยวข้อง ....

11. โรคประจำตัวอื่น ๆ ☐ไม่มี ☐มี

หากมี โปรดทำเครื่องหมาย √ ในช่อง☐ (สามารถตอบได้มากกว่า 1 ตัวเลือก)

☐ ไขมันในเลือดสูง ☐ ความดันโลหิตสูง

☐ โรคหัวใจ ☐ โรคไต

☐ โรคอื่น ๆ โปรดระบุ ....

12. ประเภทของการรักษาโรคเบาหวานที่ท่านได้รับ

☐ ไม่ใช้ยา ☐ ยารับประทาน

☐ อินซูลิน ☐ ใช้ทั้งอินซูลินและยารับประทาน

13. ประวัติการสูบบุหรี่ ☐ สูบ ☐ เคยสูบ ☐ ไม่สูบ

14. การดื่มเครื่องดื่มแอลกอฮอล์ ☐ดื่ม ☐ เคยดื่ม ☐ ไม่ดื่ม

ส่วนที่ 2 เครื่องมือที่ใช้ในการประเมินการรับรู้ความสามารถของตนเอง ,ความรู้และการจัดการตนเองในผู้ป่วยเบาหวานชนิดที่ 2

แบบประเมิน การรับรู้ความสามารถของตนเองในผู้ป่วยเบาหวาน (T-DMSES)

คำชี้แจง โปรดวงกลมรอบตัวเลขที่ตรงกับระดับความรู้สึกของท่านมากที่สุด เมื่อ 1= มั่นใจระดับน้อยที่สุด และ 5 = มั่นใจระดับมากที่สุด

ฉันมั่นใจว่า :

1. ฉันสามารถตรวจวัดระดับน้ำตาลในเลือดของฉันได้ในกรณีที่จำเป็น

1 2 3 4 5

2. ฉันสามารถลดระดับน้ำตาลในเลือดของฉันได้หากระดับน้ำตาลในเลือดของฉันสูงเกินไป เช่น เปลี่ยน) (ชนิดของอาหารที่รับประทาน

1 2 3 4 5

3. ฉันสามารถเพิ่มระดับน้ำตาลในเลือดของฉันได้หากระดับน้ำตาลในเลือดของฉันต่ำเกินไปเช่น เปลี่ยน) ชนิดของอาหารที่รับประทาน)

1 2 3 4 5

4. ฉันสามารถเลือกรับประทานอาหารที่ดีและเป็นประโยชน์ต่อสุขภาพของฉัน

1 2 3 4 5

5. ฉันสามารถเลือกอาหารที่หลากหลายโดยยังคงยึดตามแผนการรับประทานอาหารเพื่อสุขภาพ

1 2 3 4 5

6. ฉันสามารถควบคุมน้ำหนักตัวและรักษาน้ำหนักตัวให้อยู่ในระดับที่เหมาะสมได้

1 2 3 4 5

7. ฉันสามารถตรวจสอบเท้าของฉันได้ (เช่น ตรวจดูว่าควรตัดเล็บเท้าหรือไม่ หรือมีบาดแผลหรือไม่)

1 2 3 4 5

8. ฉันสามารถออกกำลังกายและทำกิจกรรมทางกายได้อย่างเพียงพอ เช่น การเดิน การเต้นแอโรบิก และการ) (ยืดเหยียดกล้ามเนื้อ

1 2 3 4 5

9. ฉันยังคงสามารถรับประทานอาหารได้ตามแผนการรับประทานอาหารเมื่อฉันป่วย

1 2 3 4 5

10. ฉันสามารถปฏิบัติตามแผนการรับประทานอาหารเพื่อสุขภาพอย่างสม่ำเสมอ

1 2 3 4 5

11. ฉันสามารถออกกำลังกายเพิ่มขึ้นได้ หากแพทย์ให้คำแนะนำว่าควรที่จะทำเช่นนั้น

1 2 3 4 5

12. เมื่อได้ออกกำลังกายเพิ่มขึ้น ฉันสามารถปรับเปลี่ยนแผนการรับประทานอาหารของตนเองได้

1 2 3 4 5

13.ฉันสามารถปฏิบัติตามแผนการรับประทานอาหารเพื่อสุขภาพได้ เมื่อฉันไม่ได้อยู่ที่บ้าน

1 2 3 4 5

14.ฉันสามารถเลือกรับประทานอาหารที่หลากหลาย โดยยึดตามแผนการรับประทานอาหาร เมื่อฉันไม่ได้อยู่ที่บ้าน

1 2 3 4 5

15.ฉันสามารถปฏิบัติตามแผนการรับประทานอาหารเพื่อสุขภาพในระหว่างช่วงที่มีงานเทศกาลต่างๆ ได้

1 2 3 4 5

16.ฉันสามารถเลือกอาหารที่หลากหลาย โดยยังคงยึดตามแผนการรับประทานอาหารเพื่อสุขภาพ เมื่อฉันต้องไปงานเลี้ยงสังสรรค์

1 2 3 4 5

17. ฉันสามารถยึดตามแผนการรับประทานอาหารของตนเอง เมื่อฉันกำลังรู้สึกเครียดหรือวิตกกังวล

1 2 3 4 5

18. ฉันสามารถไปพบแพทย์ได้ 4 ครั้งต่อ 1 ปี เพื่อทำการตรวจโรคเบาหวานของฉัน

1 2 3 4 5

19.ฉันสามารถรับประทานยาตามคำสั่งแพทย์ได้

1 2 3 4 5

20.ฉันจะสามารถรับประทานยาของฉันได้ตามปกติแม้เจ็บป่วย

1 2 3 4 5

แบบประเมิน สาระสำคัญของกิจกรรมการดูแลตนเองในผู้ป่วยโรคเบาหวาน (T-SDSCA)

คำชี้แจง คำถามต่อไปนี้จะถามท่านเกี่ยวกับกิจกรรมการดูแลตนเองในผู้ป่วยโรคเบาหวาน ในระยะ 7 วันที่ผ่านมา หากท่านป่วยในช่วง 7 วันที่ผ่านมา โปรดนึกถึงกิจกรรมการดูแลตนเองในช่วง 7 วันก่อนที่ท่านจะป่วย(โปรดวงกลมรอบตัวเลขที่ตรงกับความการปฏิบัติของท่าน)

การรับประทานอาหาร

ในช่วง 7 วันที่ผ่านมา ท่านได้ปฏิบัติตามแผนการรับประทานอาหารเพื่อสุขภาพกี่วัน ?

0 1 2 3 4 5 6 7

โดยเฉลี่ยแล้ว ช่วงเดือนที่ผ่านมา ท่านได้ปฏิบัติตามแผนการรับประทานอาหารเพื่อสุขภาพกี่วันต่อสัปดาห์ ?

0 1 2 3 4 5 6 7

ในช่วง 7 วันที่ผ่านมา ท่านได้รับประทานผักและผลไม้เท่ากับหรือมากกว่า 5 ชนิด กี่วัน

0 1 2 3 4 5 6 7

ในช่วง 7 วันที่ผ่านมา ท่านได้รับประทานอาหารที่มีไขมันสูงเช่นเนื้อแดงหรือผลิตภัณฑ์นมที่มีไขมัน กี่วัน ?

0 1 2 3 4 5 6 7

การออกกำลังกาย

ในช่วง 7 วันที่ผ่านมา ท่านได้ออกกำลังกายเป็นเวลาอย่างน้อย 30 นาที (รวมถึงการเดินออกกำลังกายอย่างต่อเนื่องเป็นจำนวนกี่วัน)

0 1 2 3 4 5 6 7

ในช่วง 7 วันที่ผ่านมา ท่านได้ออกกำลังกายในช่วงการออกกำลังกายที่เฉพาะเจาะจง เช่น เต้นแอร์โรบิควิ่ง , ปั่นจั ,เดิน ,เหยาะ ๆกรยานนอกเหนือจากที่ทำบริเวณบ้านหรือที่ทำงาน เป็นจำนวนกี่วัน ?

0 1 2 3 4 5 6 7

การตรวจระดับน้ำตาลในเลือด

ในช่วง 7 วันที่ผ่านมา ท่านได้ตรวจระดับน้ำตาลในเลือดกี่วัน ?

0 1 2 3 4 5 6 7

ในช่วง 7 วันที่ผ่านมา ท่านได้ตรวจระดับน้ำตาลในเลือด ตามคำแนะนำของบุคลากรทางด้านสุขภาพกี่วัน ?

0 1 2 3 4 5 6 7

การดูแลเท้า

ในช่วง 7 วันที่ผ่านมา ท่านได้ตรวจเช็คเท้าของท่านกี่วัน

0 1 2 3 4 5 6 7

ในช่วง 7 วันที่ผ่านมา ท่านได้ตรวจเช็คด้านในรองเท้าของท่านกี่วัน

0 1 2 3 4 5 6 7

การสูบบุหรี่

ท่านได้สูบบุหรี่ แม้แต่ครั้งเดียว (ซึ๊ดเดียว)ในช่วง 7 วันที่ผ่านมา

□ ไม่ใช่ □ ใช่

ถ้าตอบว่าใช่ โปรดระบุจำนวนมวนบุหรี่ที่ท่านสูบต่อวัน มวน ....

แพทย์ได้สั่งการรักษาใดบ้างต่อไปนี้แก่ท่าน ? โปรดระบุการรักษาทุกการรักษาที่ท่านได้รับ

□ก. ฉีดอินซูลิน 1-2 ครั้งต่อวัน

□ข. ฉีดอินซูลิน 3 ครั้งหรือมากกว่า 3 ครั้งต่อวัน

□ค. ยาเม็ดรับประทานเพื่อควบคุมระดับน้ำตาลในเลือด

□ง. การรักษาอื่น ๆ .... (โปรดระบุ)

□จ. ฉันไม่ได้รับยาตามใบสั่งแพทย์ทั้งยาเม็ดรับประทานอินซูลิน

การรับประทานอาหาร

ในช่วง 7 วันที่ผ่านมา ท่านได้งดเว้นอาหารจำพวกแป้งและน้ำตาลตลอดทั้งวัน กี่วัน

0 1 2 3 4 5 6 7

การรับประทานยา

ในช่วง 7 วันที่ผ่านมา ท่านได้ทำตามคำแนะนำแพทย์เกี่ยวกับยาโรคเบาหวานกี่วัน

0 1 2 3 4 5 6 7

ในช่วง 7 วันที่ผ่านมา ท่านได้ยาฉีดอินซูลินตามคำแนะแพทย์นำกี่วัน

0 1 2 3 4 5 6 7

ในช่วง 7 วันที่ผ่านมา ท่านได้รับประทานยาเม็ดรักษาเบาหวานตามคำแนะแพทย์กี่วัน

0 1 2 3 4 5 6 7

การดูแลเท้า

ในช่วง 7 วันที่ผ่านมา ท่านได้ล้างทำความสะอาดเท้ากี่วัน

0 1 2 3 4 5 6 7

ในช่วง 7 วันที่ผ่านมา ท่านได้แช่เท้าของท่านกี่วัน

0 1 2 3 4 5 6 7

ในช่วง 7 วันที่ผ่านมา ท่านได้ทำให้ซอกนิ้วเท้าแห้งหลังจากล้างทำความสะอาดเท้ากี่วัน ?

0 1 2 3 4 5 6 7

แบบสอบถาม ความรู้เกี่ยวกับโรคเบาหวาน (DKQ)

คำชี้แจง ข้อคำถามต่อไปนี้มีเพียงคำตอบที่ถูกเพียงข้อเดียวในแต่ละข้อคำถาม หากท่านทราบคำตอบ โปรดวงกลมรอบตัวอักษรหน้าคำตอบนั้น หากท่านไม่ทราบคำตอบ โปรดวงกลมรอบตัวอักษรหน้าตัวเลือก “ฉันไม่ทราบ”

1. สาเหตุโดยทั่วไปของโรคเบาหวานชนิดที่ 2 คือ :
   - ก. รับประทานน้ำตาลและอาหารที่หวานมากเกินไป
   - ข. อินซูลินในร่างกายทำงานไม่มีประสิทธิภาพ
   - ค. การทำงานของไตในการควบคุมน้ำตาลในปัสสาวะบกพร่อง
   - ง. ฉันไม่ทราบ
2. โรคเบาหวานที่ไม่ได้รับการรักษาโดยปกติจะทำให้น้ำตาลในเลือด:
   - ก. ปกติ
   - ข. เพิ่มขึ้น
   - ค. ลดลง
   - ง. ฉันไม่ทราบ
3. ช่วงของระดับน้ำตาลในเลือดที่ปกติคือ :
   - ก. 2.8 มิลลิโมล/ลิตร
   - ข. 6.1 มิลลิโมล/ลิตร
   - ค. 7.0 มิลลิโมล/ลิตร
   - ง. ฉันไม่ทราบ
4. ปัญหาทางสุขภาพใดต่อไปนี้<u>ไม่ได้เป็นภาวะแทรกซ้อน</u>ที่เกิดจากโรคเบาหวาน:
   - ก. โรคไต
   - ข. ปัญหาเกี่ยวกับตา
   - ค. ปัญหาเกี่ยวกับปอด
   - ง. ทุกข้อที่กล่าวมาข้างต้น
   - จ. ฉันไม่ทราบ
5. ข้อใดต่อไปนี้กล่าวถูกต้อง?
   - ก. ไม่เป็นปัญหาหากฉันไม่สามารถควบคุมเบาหวานได้เต็มที่ ตราบใดที่ฉันยังไม่มีอาการโคม่า
   - ข. เป็นเรื่องดีที่มีน้ำตาลปนกับปัสสาวะบ้างเพื่อเป็นการหลีกเลี่ยงภาวะน้ำตาลในเลือดต่ำ
   - ค. การควบคุมเบาหวานที่ไม่ดีอาจเกิดภาวะแทรกซ้อนที่รุนแรงในภายหลัง
   - ง. ฉันไม่ทราบ

6. หัวใจสำคัญของการควบคุมเบาหวานคือ :

ก. ความสมดุลระหว่างปริมาณอินซูลิน ยาเม็ดควบคุมน้ำตาล อาหาร และการออกกำลังกาย

ข. การรักษาระดับน้ำตาลในปัสสาวะให้ต่ำเพื่อป้องกันการเกิดภาวะน้ำตาลในเลือดต่ำ

ค. การรับประทานอาหารโปรตีนสูงและมีกากใยสูง

ง. ฉันไม่ทราบ

7. ผู้ที่เป็นเบาหวานควรที่จะ :

ก. ปรุงอาหารแยกจากสมาชิกคนอื่น ๆ ในครอบครัว

ข. รับประทานอาหารชนิดเดิมในเวลาเดิมทุก ๆ วัน

ค. รับประทานอาหารที่หลากหลาย สับเปลี่ยนรายการอาหารทุกวัน

ง. ฉันไม่ทราบ

8. โดยทั่วไปแล้ว ผู้ป่วยเบาหวานที่ร่างกายสมบูรณ์แข็งแรง ควรออกกำลังกาย :

ก. ครั้งละ1 ชั่วโมง สัปดาห์ละครั้ง

ข. ครั้งละ 20 -30 นาที 3-5 ครั้งต่อสัปดาห์

ค. 1 ชั่วโมง ทุก ๆ วัน

ง. ฉันไม่ทราบ

9. โดยทั่วไปการออกกำลังกายมีผลต่อการ :

ก. ลดระดับน้ำตาลในเลือด

ข. เพิ่มระดับน้ำตาลในเลือด

ค. เพิ่มน้ำตาลในปัสสาวะ

ง. ฉันไม่ทราบ

10. ข้าวส่วนใหญ่เป็น :

ก. โปรตีน

ข. คาร์โบไฮเดรต

ค. ไขมัน

ง. ฉันไม่ทราบ

11. อาหารใดต่อไปนี้ที่ท่านสามารถรับประทานได้มากเท่าที่ท่านอยากจะรับประทาน ?

ก. แอปเปิล

ข. คื่นฉ่าย, ผักชี

ค. เนื้อสัตว์

ง. ฉันไม่ทราบ

12. การตรวจสอบระดับน้ำตาลในเลือดด้วยตนเอง คือ :

ก. หัวใจสำคัญในการกำหนดปริมาณที่เหมาะสมของยา

ข. สิ่งสำคัญเพื่อดูผลลัพธ์ของการควบคุมโรคเบาหวานเช่น การรับประทานอาหารและการออกกำลังกาย

ค. ถูกทั้ง ก. และ ข .

ง. ฉันไม่ทราบ

13. ผู้ป่วยโรคเบาหวานควรที่จะดูแลเท้าของตนเองให้ดี เนื่องจาก :

ก. การได้รับยาฉีดอินซูลินที่ขาเป็นเวลานานๆ อาจทำให้เท้าบวมได้

ข. โดยทั่วไปแล้วเท้าที่มีลักษณะแบนมีความเกี่ยวข้องกับโรคเบาหวาน

ค. ผู้สูงอายุที่ป่วยเป็นเบาหวานอาจมีการไหลเวียนของเลือดบริเวณเท้าไม่ดี

ง. ฉันไม่ทราบ

14. ฤทธิ์ของยาเม็ดรับประทานรักษาเบาหวานคือ

ก. ทำให้ระดับน้ำตาลในเลือดลดลง

ข. เพิ่มการหลั่งของอินซูลิน

ค. เพิ่มการตอบสนองของอินซูลิน

ง. ถูกทุกข้อที่กล่าวมา

จ. ฉันไม่ทราบ

แบบบันทึกข้อมูลอาสาสมัคร ID Code □□□□

ผลการตรวจทางห้องปฏิบัติการและภาวะแทรกซ้อน

1. HbA1C ผลการตรวจ ………………….mg/dl

วันที่ตรวจ วัน/เดือน/ปี **(…………/…………./………….)**

2. LDL –cholesterol ผลการตรวจ…………………..mg/dl

วันที่ตรวจ วัน/เดือน/ปี **(…………/…………./………….)**

3. HDL-cholesterol ผลการตรวจ …………………..mg/dl

วันที่ตรวจ วัน/เดือน/ปี **(…………/…………./………….)**

4. Triglyceride ผลการตรวจ …………………..mg/dl

วันที่ตรวจ วัน/เดือน/ปี **(…………/…………./………….)**

5. ความดันโลหิต

5a. SBP………………. mmHg

5b. DBP………………. mmHg

วันที่ตรวจ วัน/เดือน/ปี **(…………/…………./………….)**

6. ภาวะแทรกซ้อนเรื้อรัง □ไม่มี □มี

หากตอบว่ามี โปรดทำเครื่องหมาย √ ในช่อง □ ตอบได้มากกว่า 1 ตัวเลือก

□ Cerebrovascular Accident; CVA

□ Cerebral Infraction

□ Ischemic Stroke

□ Stroke, Not specify

□ Cerebral Hemorrhage

□ Transient Ischemic Attack; TIA

□ Angina pectoris

□ Congestive Heart Failure; CHF

□ Myocardial Infraction; MI included Ischemic Heart Disease; IHD

□ Coronary Revascularization

□ Peripheral Arterial Disease, PAD

□ Neuropathy (Painful neuropathy, Autonomic neuropathy, neurogenic bladder)

□ Renal Insufficiency (CRI, CRF, CKD, ESRD)

□ Diabetes Nephropathy; DN

□ Diabetes Retinopathy; DR

□ ภาวะแทรกซ้อนอื่น ๆ โปรดระบุ……………………
