# Supplementary material for: Impact of diabetes self-management, diabetes management self-efficacy and diabetes knowledge on glycemic control in people with Type 2 Diabetes (T2D): A multi-center study in Thailand
Source: PLoS One. 2020 Dec 31;15(12):e0244692. doi: 10.1371/journal.pone.0244692 (PMC7774854; doi:10.1371/journal.pone.0244692)
Supplement: Attachment — Submitted filename: Response to Reviewers.docx [file pone.0244692.s003.docx]

Dear Prof Chung-Ying Lin

We thank you for the opportunity to resubmit our manuscript *Impact of diabetes self-management, diabetes management self-efficacy and diabetes knowledge on glycemic control in Type 2 Diabetes (T2D) patients: A prospective multi-center study in Thailand.* (PONE-D-20-18140) to PLOS ONE. Detailed below is each editorial and reviewer comment, our response, and where appropriate, the action taken. Please note that our comments are listed by editorial comments (EC), and those from reviewer 1 (R1) and reviewer 2 (R2), respectively.

| **Comment** | **Response/Action** |
| --- | --- |
| **EC-1:** I would like the authors to acknowledge that Hong Kong has implemented a Patient Empowerment Programme (PEP) on people with type 2 diabetes. The PEP is based on the concept of self-efficacy and has been found to be effective on self-care behaviors and health outcomes among people with type 2 diabetes. Therefore, the authors should discuss the PEP in their paper (then 5 references are provided) | Thank you for this. After reading these papers we were very impressed with the inroads made with the PEP in Hong-Kong. We have now included the following test in the main part of the discussion (regarding the interplay between diabetes knowledge, management self-efficacy and self-management):  ACTION: Added text: *This is a question that has been investigated, albeit indirectly, in a study of the mechanisms underpinning patient diabetes self-care behaviour, and whether a Patient-Empowerment Programme (an intervention addressing both knowledge and attitude among people with T2D) may improve self-care behaviour in people with diabetes in Hong Kong[48]. Indeed the PEP was subsequently shown to improve clinical targets and processes of care [49], longer term patient outcomes including mortality 50] and health-related quality of life [51] in this population. However, the complex interplay between Diabetes knowledge, management self-efficacy and self-management still needs further investigation.*  Please note: The above text is also used to address the 11^th^ (final) comment from Reviewer 2. |
| **EC-2:** In your Methods section, please provide additional information about the participant recruitment method and the demographic details of your participants. Please ensure you have provided sufficient details to replicate the analyses such as: a) the recruitment date range (month and year), b) a description of any inclusion/exclusion criteria that were applied to participant recruitment, c) a description of how participants were recruited, and d) descriptions of the specific locations where participants were recruited and where the research took place. | Both a) and b) were already stated in the original manuscript: *Questionnaires were administered in February to June, 2016 to T2D outpatients aged at least 20 years old who had had a diagnosis of T2D for at least 3 years, able to read and understand the Thai language and were willing to participate in the study.*  ACTIONS:  For c) we have now added the text: *Potential participants were identified by the receptionist nurse upon arrival for their routine diabetes check-up. These patients were approached while waiting for their appointment and asked if they would participate. Of all of the patients approached (708) all agreed to participate, but eight patients were excluded as they were not fluent in the Thai language.*  For d) we have now added the text: *The four hospitals from which participants were enrolled were the Phupaman community hospital in Khon Kaen, Srinagarind hospital at Khon Kaen University, and Wechkaroonrasm community hospital and the King Chulalongkorn Memorial Hospital (Chulalongkorn University) which are both located in Bangkok.* |
| **EC-3:** Please provide a sample size and power calculation in the Methods, or discuss the reasons for not performing one before study initiation. | As this study was an observational study (not an RCT) with three continuous effects of interest (DK, DMSE and DSM) along with a large set of additional covariates (which may be either independent risk factors or confounders), there is no formal sample size formula that is appropriate for such a situation. The only way to estimate sample size in such a situation is via simulation (which involves speculation about the effect sizes) or an informal sample size calculation such as the 10EPV approach (Harell *et al*, 1996). We did use th 10EPV approach despite its usual purpose being for sample size estimation in multivariable prognostic studies (where emphasis is on prediction, rather than the power of hypothesis tests.  ACTION: We have added the following text to the first subsection of the methods:  *As this was an observational study with three study effects (Diabetes Knowledge, management self-efficacy and self-management) and a large number of potential covariates we used the 10 cases per predictor approach advocated by Harell and colleagues (1996)[19] to estimate our sample size. Assuming a prevalence of blood glucose control of 0.36 [20] and up to 25 parameters in the final model, we estimated a sample size of 695 patients (rounded up to 700) was required.* |
| **EC-4:** Please include additional information regarding the survey or questionnaire used in the study and ensure that you have provided sufficient details that others could replicate the analyses. For instance, if you developed a questionnaire as part of this study and it is not under a copyright more restrictive than CC-BY, please include a copy, in both the original language and English, as Supporting Information. | ACTION: We have now included an English and Thai version of the survey instrument as supporting information. |
| **EC-5**: We note that you have indicated that data from this study are available upon request. PLOS only allows data to be available upon request if there are legal or ethical restrictions on sharing data publicly. | ACTION: We have now uploaded a de-identified version data along with a data dictionary to the Mendeley data repository. We have now added the following reference:  Hurst, Cameron (2020), “Type 2 diabetes and Psychosocial determinants of achieving clinical targets”, Mendeley Data, V1, doi: 10.17632/xhmjcfp2ym.1  We have also updated our cover letter accordingly. |
| **REVIEWER 1** | |
| **R1-1**: This is NOT a prospective study- please change the title. | We do admit this is an ambiguous term in the context of this study design. However, we would like to point out that ALL the main were collected prospectively and for the purposes of the study including the blood sugar control outcome (which, we admit, was extracted from the medical records, but for that day’s visit). However, we do admit some of the secondary variables (e.g. complications and comordbidities) are retrospective as they were collected from the patients’ medical records. We have now excluded the term not so much because we believe it is inaccurate but because people commonly confuse the term ‘prospective’ with ‘prospective cohort’ (where the word prospective usually occurs). Nowhere do we claim this study is a cohort study.  ACTION: We have now removed the word ‘prospective’ from the study design from the only place it occurred (in the title) |
| **R1-2:** Methods are not complete. How were the subjects recruited? Were they approached when they were waiting for an office visit? After the visit? Were the subjects approached in their homes? By mail? By phone? How many of the subjects approached agreed to participate? What are the inclusion and exclusion criteria? Did you have to not include any subjects? What were the reasons the subjects were not eligible? What is meant by the patients were recruited until the study was completed? | We address all of these issues in Editor comment 2  However, we feel we need to provide an answer to the last question (What is meant by the patients were recruited until the study was completed?) This one slipped past us. We meant to say until the required sample size was reached. We have now excluded this comment and added clarification (see response to Editor comment 2c). |
| **R1-3**: If the de-identified data are available by request from author NR, why aren’t these data freely available without restrictions? | We have now uploaded de-identified data and the data dictionary to the Mendeley data repository and added a reference. See EC-5 above. |
| **R1-4**: Lines 67-70 states that the increasing prevalence of T2DM is mainly due to an ageing population. Reference #2 does not say that. I do not think that is the cause of increasing prevalence. If there is a reference to support that statement, please provide. | We would like to point that reference #2 is referring to the following sentence (about 80% of diabetes cases living in low and middle income countries) but we take your point. We have now amended this sentence:  ACTION: Amended sentence to read: *Approximately 90% of patients with diabetes have Type 2 Diabetes Mellitus (T2D) and the increasing prevalence of T2D is likely to be attributable to factors such as ageing populations and an increasing level of sedentary life styles that seem to accompany economic development.* |
| **R1-5**: Lines #105-109- please remove “for example” next to your references. | ACTION: We have now made this amendment. |
| **REVIEWER 2** | |
| **R2-1:** Data availability- As I read the journal instructions, having the data available through contact with an author is not an acceptable alternative. The authors explain that the original dataset has identifiable information, so it is OK to restrict access to that. But the authors worked with a deidentified dataset, so I don’t think that author contact is acceptable. But this is a point for the journal editors to address. | We have now uploaded de-identified data and the data dictionary to the Mendeley data repository and added a reference. See EC-5 above. |
| **R2-2:** The Abstract should have a sentence or two describing the reasons for doing this study and predetermined hypotheses. | Actually, we think our opening sentence in the abstract: *We investigate the relationship of diabetes knowledge, diabetes management self-efficacy and diabetes self-management with blood glucose control among people with Thai type 2 diabetes mellitus (T2D).* Makes it clear that what our motivation for the study is. However, we |
| **R2-3:** Line 68- While aging of the population certainly contributes to increasing prevalence, I don’t think you can say that is the “main” cause- rising rates of obesity and sedentary lifestyle are also factors (which I see that you note in the following paragraph). | ACTION: We have now changed this statement. Please see Reviewer 1-Comment 4. |
| **R2-4:** Line 96- Most medical journals use the term “blood glucose,” not “blood sugar,” which is a more colloquial term. Similarly, most diabetes journals no longer accept “diabetes patient,” as it is deemed to be stigmatizing. Instead one writes “person with diabetes,” some even shorten that to “PWD.” These are minor quibbles, but making these changes throughout the manuscript would conform to currently accepted terminology. | ACTION: We have now substituted:   a) *blood glucose* for *blood sugar*; and b) *People with Type 2 diabetes* for *Type 2 diabetes patients*   Throughout the entire manuscript. |
| **R2-5:** Please say a little more about recruitment procedures, i.e., how were potential participants approached. Of those approached, how many refused? Whle the fact that those who consented did complete the questionnaires is good, the reader also needs to know something about those who refused, to address generalizabiity of the results. Also, you state that data were gathered until it was complete, meaning that you had 700 participants. How did you determine that you needed 700 participants? | All of these issues are addressed in Editorial comment number 2 (**EC-2**) |
| **R2-6:** Line 135- It’s Toobert, not Toober. | ACTION: We have now corrected this typographical error. |
| **R2-7:** Very nice work validating the measures for use in a sample of Thai individuals. | We thank the reviewer |
| **R2-8:** Why did you choose to look at A1c as a dichotomous variable? You lose a lot of data points by doing so, as we know that an A1c of 7.0% isn’t very different than one of 7.1%, yet the former is a failure and the latter is a success. Please either perform longitudinal analyses, or explain why a dichotomous approach is appropriate. | JUSTIFICATION: Actually as both a biostatistician and clinical epidemiologist, the reviewer’s comment is music to my ears. I wholeheartedly agree with their sentiment. The use of discretised clinical measures (especially in the absence of a strong threshold effect) is, from a statistical point of view, highly inadvisable (the wide use of BMI classes is probably one of the best examples). In the case of HbA1c, using a binary ‘control’ variable groups together is likely to group highly disparate individuals on the one hand (a patient with a1c = 7.1% and one with a1c of 13% are considered the same), and on the other hand, separates very similar patients (e.g. a patient with 7.0% and one with a1c = 7.1% are considered different). The likely result of this is to weaken associations.  However, having said this, many clinicians and policy makers want to class people as it helps in following (in the case of the former) and developing (in the case of the latter) guidelines. That is, there are often very practical reasons for the discretization of continuous clinical measures. This is particularly true in the health services research setting. In Thailand (and many other places) uncontrolled blood glucose is classed as A1c > 7.0%.  Another disadvantage of analysing continuous outcomes (one I have noticed thousands of times over the course of my careers) is that researchers and clinicians often over-rely on p-values when they are presented with mean differences on scales to which they have often given insufficient thought. With a study involving 700 patients it is likely that such comparisons would be highly overpowered meaning it is possible (and even likely) that trivial differences will be identified as statistically different, and then misread as clinically important. We admit that in an cross-sectional study such as this that sample size calculations cannot be even close to exact, so reinforcing both clinical importance (makes a difference to patient outcomes) and statistical significance (effect unlikely to have been observed by chance) is of critical importance. This is something we don’t need to do in the simpler designs (e.g. RCTs and pre-post tests) where a formal sample size calculation can be done to align clinical and statistical significance.  A final potential disadvantage to continuous clinical outcomes is non-linearity. The fact is that a difference in HbA1c of 0.25 may be less (or more) important when comparing HbA1c of 7.0% to 7.25% than when comparing 11.0% to 11.25%. These subtlies are often ignored by many researchers and clinicians when reading the research of others.  So in summary our real reason for using discretised version of a1c (controlled vs uncontrolled) is pragmatic (and from bitter experience). In other words, we felt our work would get much better traction where we used a standard indictor of control (which, for most people, is much more comparable across studies) then a continuous version of blood glucose control. Whenever I have used continuous versions of variables (where discrete versions are usually used) I have almost invariably been criticized by reviewers. While methodologically the use of discretised measures (in the absence of a threshold effect) is not a good idea, I do see where their use might make it easier for building the evidence for changing practice. |
| **R2-9:** Table 2- Suggest you create a simpler title, and put info about the different models as footnotes- very hard to read. | I can only assume that the reviewer is referring to Table 3 which is the long-winded one (Table 2’s title is rather standard).  ACTION: We have now changed  1.Table3’s legend to:  ***Table 3 Four different models of blood-glucose control which vary in terms of the combinations of the diabetes knowledge, management self-efficacy and self-management constructs included. All four models are also adjusted for all other patient characteristics.***  And  2. added the following list (of the four models) to the footnote:  *Model 1 Effect of Diabetes self-management adjusted only for patient characteristics,*  *Model 2a Effect of Diabetes self-management adjusted for diabetes management self-efficacy and patient characteristics,*  *Model 2b Effect of Diabetes self-management adjusted for diabetes knowledge and patient characteristics, and*  *Model 3 Effect of Diabetes self-management adjusted for both diabetes management self-efficacy, diabetes knowledge along with patient characteristics* |
| **R2-10:** Line 324- The statement that interventions should not focus on diabetes knowledge, since knowledge didn’t relate to glycemic control, is an over-reach. Given the cross-sectional nature of the data, you might find that knowledge does predict A1c longitudinally. Also, there are other benefits to knowledge, and I’d assume that high knowledge relates to high self-efficacy. I may have missed it, but did you look at the relationships between the measures? If not, you should, as it might clarify what you’ve found. | I found this to be a particularly insightful comment and you are right to bring this up. However, to be fair, we don’t say that interventions should not include a diabetes knowledge component, rather that interventions which predominantly or solely focus on diabetes knowledge may not may not be as effective as interventions that also try to directly improve patient’s attitude (e.g. self-efficacy) and practice (i.e. self-care). However, the fact that the reviewer raises this point my mean we need to provide further clarification. We do this by adding an example where adding an ‘attitude and practice’ component to an intervention significantly improved patients’ self-care behaviour. Specifically,  ACTION: We have now added the following text to the end of the paragraph: *Regardless, the lack of association between diabetes knowledge and glycaemic control among Thai people with T2D means we may need to reconsider early educational interventions in Thais with T2D which have a strong emphasis of knowledge transference [43], and typically do not have components which directly focus improving patients’ attitude or self-care behaviour. Indeed, a study of the impact of an intervention in Hong Kong people with diabetes demonstrated considerably better post-intervention self-care in participants who also completed the ‘empowerment’ component of the program compared to those that did the disease knowledge component alone.[48]*  We also acknowledge that our design makes in difficult to tease out the true impact of diabetes knowledge on blood glucose control and that the lack of association we observed may be due to reverse causality (patients who have had the disease longer, and consequently have poorer control, are likely to have better knowledge). Indeed we already acknowledge this earlier in the paragraph. |
| **R2-11:** The diabetes self-efficacy discussion is a little convoluted. It certainly makes sense that people who manage their diabetes well also have a high level of self-efficacy, i.e., they believe they can do it because they are doing it (or they are doing it because they believe they can do it- who knows?). The notion that you should target self-efficacy, as if it’s separate from actual self-management has always troubled, and confused, me. I don’t think self-efficacy is “largely ignored” in interventions, I think it’s just assumed that improving knowledge, and improving self-care, will result in improving self-efficacy too. I’m not sure what changes I’d recommend, but these conclusions need attention. Perhaps future research needs to take a more longitudinal approach to test out the hypotheses I’ve stated. | There are several comments here and I will address them separately:   1. The diabetes self-efficacy discussion is convoluted:   Actually we took great pains with this particular paragraph. The fact is that unpacking the complex interplay between diabetes knowledge, management self-efficacy and self-management and how these, both collectively and individually, contribute to clinical targets, processes of care and longer term patient outcomes, given our current knowledge, is very difficult. The role of this paragraph is to convey this complexity and to acknowledge that our study had design limitations which make it difficult to make inferences about these relationships. I redrafted this paragraph many times in the original manuscript in an attempt to make it as clear as possible, but the fact remains that the interrelationships between these components are complex, and any subsequent discussion of the relationships, to do the problem justice, will of necessity, appear convoluted.   2. Targeting self-efficacy (as if it is separate from Self-management) in an intervention.   It seems that the reviewer is looking at this from an intervention point of view, rather than how the self-efficacy may manifest itself in patients as either a result of learned (or reinforced) behaviour, or whether diabetes management self-efficacy may also, or instead, be driven by a latent trait. The answer to this question must surely have bearing on how we design our interventions. It is also not a question we feel that we can answer (given the limitations imposed by our study design, or the work done by anybody else), but we do pose this important question nonetheless. I also admit that in some interventions, some thought has been given to, or at least assumptions made about, the role that self-care ‘practice’ may have on patients’ disease management self-efficacy. However, in Thailand the focus in interventions has been solely on diabetes knowledge, and not attitude, nor practice. The reviewer should note that our statements regarding current interventions is mainly confined to the current practice in Thailand. We acknowledge that more sophisticated interventions have been trial elsewhere and indeed we have now added the following text to clarify this:  ACTION: We have now added the following text to clarify this point (btw we also allude to this text in EC-comment 1):  *This is a question that has been investigated, albeit indirectly, in a study of the mechanisms underpinning patient diabetes self-care behaviour, and whether a Patient-Empowerment Programme (an intervention addressing both knowledge and attitude among people with T2D) may improve self-care behaviour in people with diabetes in Hong Kong[48]. Indeed the PEP was subsequently shown to improve clinical targets and processes of care [49], longer term patient outcomes including mortality 50] and health-related quality of life [51] in this population. However, the complex interplay between Diabetes knowledge, management self-efficacy and self-management still needs further investigation.* |
